# Reinstating respiratory heart rate variability improves hemodynamic responses during exercise in heart failure with reduced ejection fraction

**DOI:** 10.1007/s00395-025-01110-3

**Published:** 2025-05-03

**Authors:** Julia Shanks, Mridula Pachen, Nigel A. Lever, Julian F. R. Paton, Rohit Ramchandra

**Affiliations:** 1 Department of Physiology, Faculty of Medical and Health Sciences, Manaaki Manawa – The Centre for Heart Research, University of Auckland, 85 Park Road, Grafton, 1023 Auckland, New Zealand; 2 Department of Cardiology, Auckland City Hospital, Auckland District Health Board, Park Road, Grafton, Auckland, New Zealand

**Keywords:** Heart failure, Respiratory heart rate variability (RespHRV), Cardiac output, Coronary artery blood flow, Pre-clinical, Exercise, Sheep

## Abstract

Individuals with heart failure have significantly reduced exercise capacity, a critical life-limiting symptom for those living with the disease. Heart failure is negatively correlated with decreased heart rate variability, including the loss of heart rate variability in tune with breathing—termed respiratory heart rate variability (RespHRV). We tested the hypothesis that restoration of RespHRV would improve exercise tolerance. Heart failure was induced in adult female sheep using a microembolization technique, and the sheep were divided into two groups: RespHRV paced and monotonically paced. Following a 1-week baseline recording, the sheep underwent 2 weeks of pacing. Direct recordings of hemodynamic parameters, including arterial pressure, cardiac output, coronary artery blood flow, and heart rate, were taken at rest and during treadmill exercise. Reinstating RespHRV significantly increased resting cardiac output, a change not observed in monotonically paced sheep. Neither group showed a change in resting coronary artery blood flow. During exercise, RespHRV-paced sheep showed increased cardiac output, coronary artery blood flow, cardiac power output, and faster heart rate recovery post-exercise. In contrast, monotonically paced sheep showed no changes in exercise-induced cardiac function. A separate group of heart failure animals were studied to determine if these benefits would persist alongside heart failure medications. RespHRV pacing continued to improve resting cardiac output with concurrent heart failure medications. Our results indicate that reinstating RespHRV may be a novel approach for improving outcomes in heart failure, including exercise capacity.

**Supplementary Information:**

The online version contains supplementary material available at 10.1007/s00395-025-01110-3.

## Introduction

Heart failure is a pressing global concern, affecting an estimated 64 million individuals worldwide [49]. The prevalence of heart failure continues to rise [12], imposing substantial economic burdens on both individuals and society due to direct costs of hospital care and medication, as well as indirect costs such as decreased productivity [24]. Chronic heart failure with reduced ejection fraction (HFrEF) is characterized by diminished left ventricular pump function, leading to decreased exercise tolerance [57], autonomic imbalance [17, 20], and a significant decline in the quality of life [25]. In healthy individuals, the heartbeat naturally varies with every breath (called respiratory heart rate variability, abbreviated as RespHRV). However, in patients with HFrEF, RespHRV is lost [23], which is a negative prognostic indicator of cardiovascular diseases[ 56]. Over the last 2 decades, pacemakers which are used to restore heart rhythm have evolved to more closely mimic cardiac physiology[ 1, 2, 55]. While this has improved outcomes in patients, these devices still have limitations[ 15] and no commercially available pacemaker can currently reinstate physiological RespHRV. The overarching aim of this study was to examine how reinstatement of RespHRV would alter heart function in HFrEF.

During acute demands on the heart, such as exercise, coronary artery blood flow increases to respond to the increased oxygen demand of the working myocardium [35]. An early symptom of HFrEF is reduced cardiac capacity during exercise, including impaired coronary artery blood flow, which is directly linked to myocardial dysfunction [26]; all this contributes significantly to reduced quality of life in patients with HFrEF [45]. Patients also experience cardiac vagal autonomic dysfunction, which is observed in delayed post-exercise heart rate recovery [3, 5, 29, 37]. While RespHRV pacing improves cardiac output at rest [51], its ability to adapt cardiac function to meet the increased energy demands during exercise is unknown and was the primary aim of the present study. We also assessed whether heart rate recovery post-exercise, a hallmark indicator of cardiovascular fitness, would be improved with RespHRV pacing.

Before clinical translation, it is crucial to demonstrate the efficacy of any treatment against a backdrop of current medications. To determine if the beneficial effects of RespHRV would persist with medications, we also examined the response to reinstatement of RespHRV against a background of β-blocker and angiotensin AT_1_R blocker therapy in a separate group of animals. Our study was specifically designed to investigate the clinical impact of reinstating RespHRV in chronic heart failure, where the loss of RespHRV is correlated to increased adverse outcomes.

## Methods

### Sheep

All animal studies and surgical procedures followed relevant guidelines and were approved by the Animal Ethics Committee of the University of Auckland (#2082). Adult (3–6 year old) female Romney sheep (*n* = 32 total) were sourced from Ngāpouri Liggins research farm and housed in individual crates at the University of Auckland, large animal unit. All sheep used were novel for this study and not included in our previous publication. Sheep were acclimatized to laboratory conditions (18 °C, 50% relative humidity, 12-h light–dark cycle) and human contact for 1 week before any experiments. Sheep were fed 2–2.5 kg/day (Country harvest pellets) and had access to water ad libitum. Male sheep (rams) are often unpredictable and aggressive, making measuring echocardiography and stable hemodynamics difficult in conscious animals. Although we anticipate that the results obtained from this study will apply to both males and females, we acknowledge that the absence of males is a limitation of this study.

**Fig. 1:**
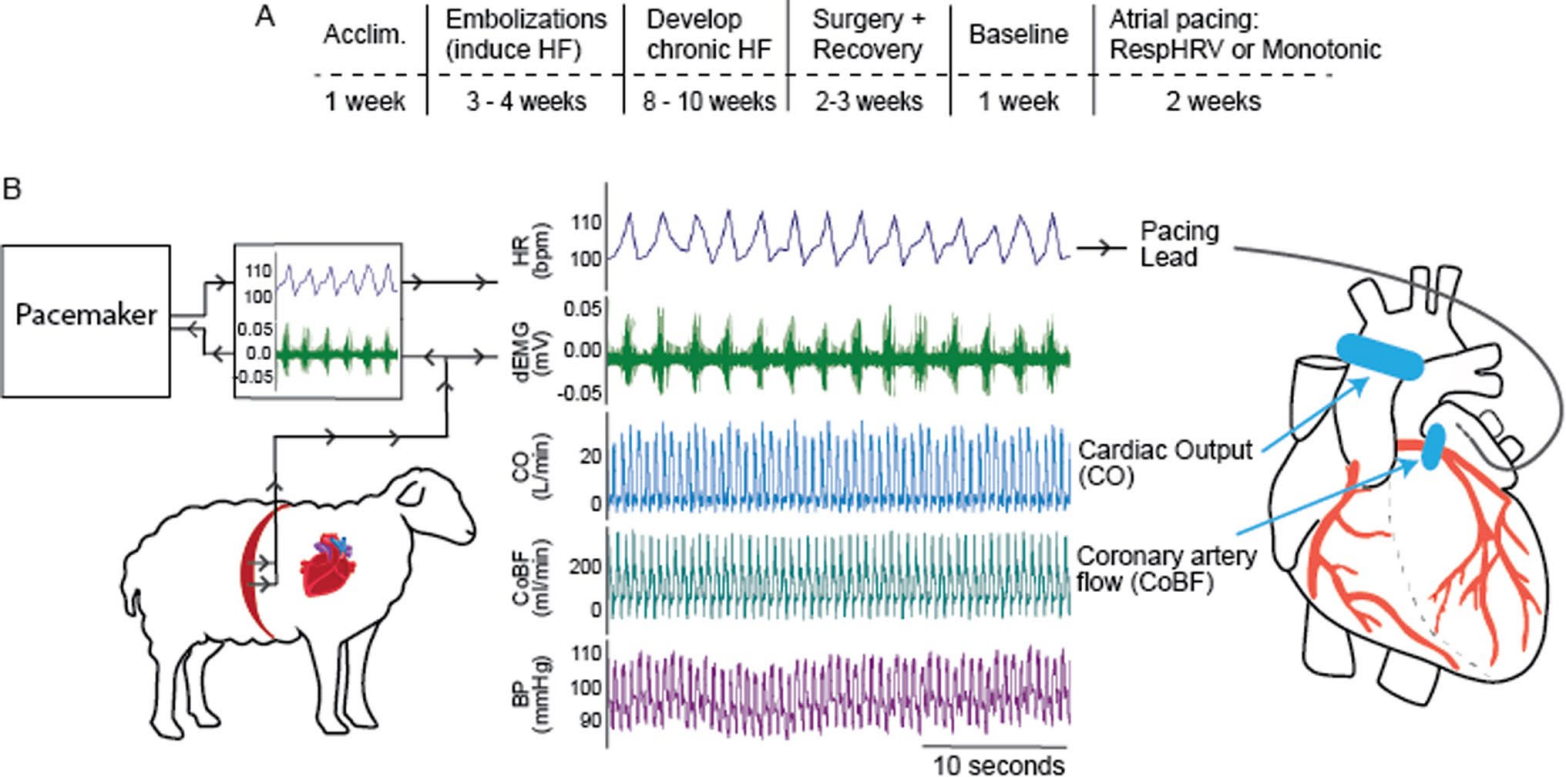
Experimental set-up. **A**, Timeline of experimental protocol. **B**, Schematic of the experimental set-up and representative simultaneous recordings in awake sheep during RespHRV pacing. Blue ovals on the heart schematic indicate the placement positions of implanted flow probes. BP; blood pressure, CO; cardiac output, CoBF; coronary artery blood flow, dEMG; diaphragmatic electromyography (edited to remove ECG contamination for visual presentation), HR; heart rate

Two protocols were used: The first 16-week protocol is described below; a summary is schematized in Fig. 1A (Protocol A). The second 18-week protocol, which included heart failure medication (Protocol B), is described later in the Methods section. This study focused on observing the benefits of reinstating RespHRV compared to non-respiratory modulated (monotonic) pacing in heart failure; studying RespHRV pacing in healthy animals was irrelevant to this study.

### Data availability

The data that support the findings of this study are available from the corresponding author upon reasonable request.

### Induction of heart failure: embolization surgical procedure

To determine the effects of RespHRV pacing on measures of exercise capacity (Protocol A), two groups of heart failure sheep were studied: respiratory HRV (RespHRV: *n* = 9) and monotonically (mono: *n* = 10) paced. To determine if the effects of RespHRV pacing persisted with medications (Protocol B), a separate group of RespHRV animals (*n* = 8) were studied. In both protocols, a microembolization technique was used to induce reduced ejection fraction heart failure as previously described [3, 51]. In brief, sheep were anaesthetized with an induction by 2% Diprivan (Propofol) (5 mg/kg i.v. AstraZeneca, AUS), maintained with a 2% isoflurane-air-O_2_ mixture and were intubated for mechanical ventilation. Anesthesia depth was monitored throughout the surgery by an absence of the corneal reflex and an absence of a withdrawal response to a noxious pinch. One femoral artery was accessed percutaneously using an 8F (CORDIS®, USA) sheath and under fluoroscopic guidance, the catheter was progressed into the left main coronary. Prior to the injection of microspheres, β-blocker (metoprolol up to 20 mg/kg, IV) and lignocaine (2 mg/kg, IV) were injected intravenously to prevent ventricular arrhythmias. Polystyrene latex microspheres (45 μm diameter; 1.4 mL, Polysciences, Warrington, PA, USA) were injected into the coronary artery to induce ischemic heart failure. Following recovery for a week, up to two more embolizations were conducted until the ejection fraction dropped to our target value (~ 45%).

Conscious sheep underwent echocardiography (GE S70); ejection fraction dropped from 60–70% (pre-embolization) to ~ 45% (3 days post-embolization). Echocardiograms were repeated 3 months post-embolization to confirm sheep were in heart failure. In the short-axis M-mode, diastole, systole, and ejection fraction were obtained and calculated for the left ventricle.

A total of *n* = 32 sheep underwent microembolization surgery, *n* = 3 sheep did not reach the ejection fraction cut-off criteria for heart failure and were excluded from the study. For the first protocol, the remaining sheep were randomly assigned to the RespHRV or the monotonic groups. One sheep in each of the monotonic- and RespHRV-paced groups did not maintain stable pacing with high enough efficacy [51] across the 2-week pacing period due to lack of contact of the pacing lead and were excluded from the analysis. This resulted in data being collected and analyzed in RespHRV-paced (*n* = 9) and monotonically paced (*n* = 10) sheep. For the second protocol (Protocol B), two sheep lost cardiac output or respiratory signals during the protocol so were excluded and this resulted in *n* = 6 animals in this group. No sheep died during either of the pacing protocols.

### Instrumentation surgery

Following induction (Propofol, 2% Diprivan. 5 mg/kg i.v., AstraZeneca, AUS) and maintenance (2–3% isoflurane-air-O_2_ mixture) of anesthesia, sheep were placed on their right side, and instrumentation was carried out in sterile conditions. A 10 cm incision was made on the left side of the neck. A single-tip pressure probe (Millar Inc., Texas, USA. Model # 320–6590) was inserted into the left common carotid artery to get an index of blood pressure, and a cannula was inserted into the left jugular vein for drug infusion. The pressure probe and catheter were secured with a purse string suture (Filasilk, 3.0 non-absorbable braided silk suture) to maintain blood flow through the vessel.

An intercostal nerve block was performed by injecting bupivacaine (0.25%. Aspen, New Zealand) into the second, third, fourth, fifth, and sixth intercostal spaces (7.5 µg per injection, ~ 0.625 µg/kg total). Anesthesia depth was routinely monitored throughout the surgery by checking the response to pinching the hoof and the response to eye-lid contact. If necessary, the level of isoflurane was altered (within the 2–3% range). A dorsal to ventral incision was made on the left side of the chest, and the fourth rib was removed to access the heart. A Doppler flow probe (Size 28, Transonic, AU) was placed around the ascending aorta to measure directly beat-to-beat cardiac output. A Doppler flow probe (Size 6, Transonic, AU) was placed around the left main coronary artery to measure coronary artery blood flow. For cardiac pacing, two pacing leads (Biotonik, Berlin, Germany, Solia S 53; in case of failure in one) were secured externally to the left atrium with 3.0 Filasilk suture and insulated in silicone gel. To gain a measure of respiration, electrodes were implanted into the diaphragm to measure diaphragmatic EMG ('DEMG') as an index of inspiration as previously described (Fig. 1B) [51].

Flow probes were tunneled subcutaneously and exited percutaneously on the dorsum of the sheep to connect to chronic, continuous recording devices after recovery. Sheep were given antibiotic injections (20 mg /kg intra-muscular; oxytetracycline, Phoenix, NZ) and analgesia (ketoprofen 10%, 2 mg/kg intra-muscular; Merial, Boehringer Ingelheim, NZ) at the start of surgery, and for the first 3 days postoperatively. Animals were allowed to recover for 7 days post-surgery before beginning the exercise acclimatization protocol. All parameters were recorded from conscious sheep on a desktop computer with a CED Micro-1401 interface and a data acquisition program (Spike2 v8, Cambridge Electronic Design, UK).

### Hemodynamic measurements and analysis

A baseline recording period was acquired when heart rate and cardiac output had stabilized postoperatively (between 5 and 7 days. Table 1). Continuous arterial blood pressure, cardiac output, and coronary artery flow were recorded 24/7 for around 6 weeks, all sampled at 1000 Hz. Heart rate was calculated from the inter-pulse interval of the blood flow in the ascending aorta. DEMG signal was amplified (X10, 000) and filtered (band pass 0.3–3.0 kHz).

**Table 1:** Raw values for resting hemodynamic parameters in conscious free-standing sheep that underwent the exercise protocol (Protocol A): 24-h averages

	Baseline (not paced)		1-week pacing		2-week pacing	
	RespHRV (pre-pace)	Mono (pre-pace)	RespHRV (paced)	Mono (paced)	RespHRV (paced)	Mono (paced)
Heart rate (bpm) RespHRV, *n* = 6 Mono, *n* = 8	97 ± 5.5	98 ± 5.5	112 ± 6.7	112 ± 5.1	113 ± 5.4	114 ± 4.6
Cardiac output (L/min) RespHRV, *n* = 6 Mono, *n* = 8	8.0 ± 0.9	7.5 ± 0.5	9.2 ± 0.9	7.4 ± 0.5	9.7 ± 0.9	7.4 ± 0.6
Coronary artery blood flow (ml/min) RespHRV, *n* = 5 Mono, *n* = 5	95 ± 16	77 ± 12	97 ± 13	77 ± 16	101 ± 17	73 ± 15
Mean arterial pressure (mmHg) RespHRV, *n* = 5 Mono, *n* = 6	83 ± 3.3	84 ± 2.1	90 ± 7.9	83 ± 3.6	81 ± 3.6	80 ± 4.0

### Pacing Protocol A

**Fig. 2:**
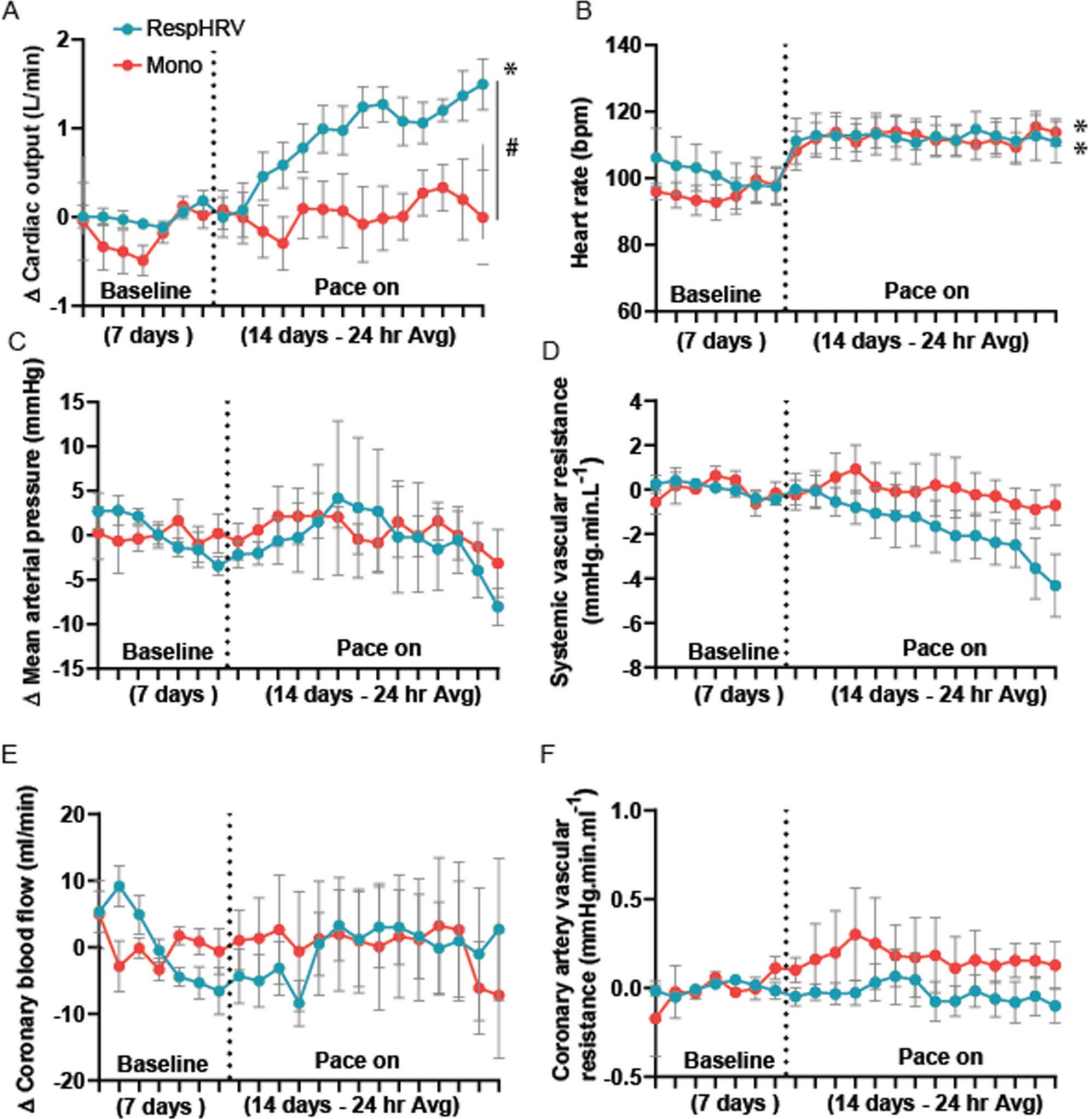
Conscious 24-h data for 3-week protocol. **A**, Two weeks of RespHRV pacing (blue: *n* = 6) significantly increased cardiac output compared to monotonic pacing (red: *n* = 8) (**A**, RespHRV: * *P* = 4.82 × 10^–6^. One-way ANOVA, effect of time. # *P* = 0.0065. Two-way ANOVA, interaction effect). **B**, Both RespHRV and mono groups were paced to an equivalent heart rate (RespHRV: * *P* = 0.027, *n* = 6, Mono: * *P* = 2.68 × 10^–6^, *n* = 8 One-way ANOVA, effect of time). No change in **C**, mean arterial pressure (RespHRV: *n* = 5, Mono: *n* = 6), **D**, systemic vascular resistance (RespHRV: *n* = 5, Mono: *n* = 6), **E**, coronary artery blood flow (both groups, *n* = 5), or **F**. coronary artery vascular resistance (RespHRV: *n* = 5, Mono: *n* = 4) between 2 weeks RespHRV or monotonic pacing. **A**–**F**, Each data point is a 24-h average

The cardiac pacing was set at 10–15 beats per minute above the resting heart rate of each sheep, with stimulation parameters of 1.5–2.5 V 2 ms pulse width (Fig. 2C). Pacing voltage was increased if pacing became intermittent during the protocol. For the monotonically (Mono) paced group (*n* = 10), pacing leads were connected to a stimulator (Grass Instruments). For the RespHRV group (*n* = 9), RespHRV pacing was achieved using a biofeedback device described previously [40–42], with an RespHRV magnitude (peak-to-trough) of 12 beats per minute; this was optimized previously [7, 51]. RespHRV pacing was visually checked at least once daily against the DEMG channel to ensure the rising phase of heart rate correlated with inspiration and the falling phase with expiration (Fig. 1B).

### Pacing Protocol B

The cardiac pacing in this group was also set at 10–15 beats per minute above the resting heart rate of each sheep, with stimulation parameters of 1.5–2.5 V 2 ms pulse width. Pacing voltage was increased if pacing became intermittent during the protocol. RespHRV pacing was achieved using the same biofeedback device as Protocol A with an RespHRV magnitude (peak-to-trough) of 12 beats per minute. RespHRV pacing was visually checked at least once daily against the DEMG channel to ensure the rising phase of heart rate correlated with inspiration and the falling phase with expiration.

### Pacing efficacy

Pacing efficacy was calculated to determine the percentage of the day (24 h) the animals were being paced. For monotonically paced sheep, the number of total heartbeats versus those at an R-R interval outside the target paced range was calculated as a percentage for 24 h. For RespHRV-paced animals, a threshold horizontal cursor was placed on the heart rate channel below the pre-set peak heart rate change during inspiration. For a 24-h period, the number of heartbeats that were modulated by the pacemaker was divided by the number of breaths calculated from the dEMG signal and converted to a percentage to give RespHRV pacing efficacy [51]. ‘Loss’ of RespHRV pacing usually occurred over extended periods (mins) when intrinsic heart rate rose above the RespHRV threshold. To be included in the RespHRV-paced group, pacing efficacy needed to be ≥ 35% of all breaths for the average 24-h period, as previously reported [51], and mimicked in clinical conditions where RespHRV is most prominent during non-REM sleep and lost during activity [4, 39]. When not respiratory modulated pacing, the RespHRV-paced group was paced at a fixed rate equivalent to the monotonic-paced group.

### Exercise test in Protocol A

**Fig. 3:**
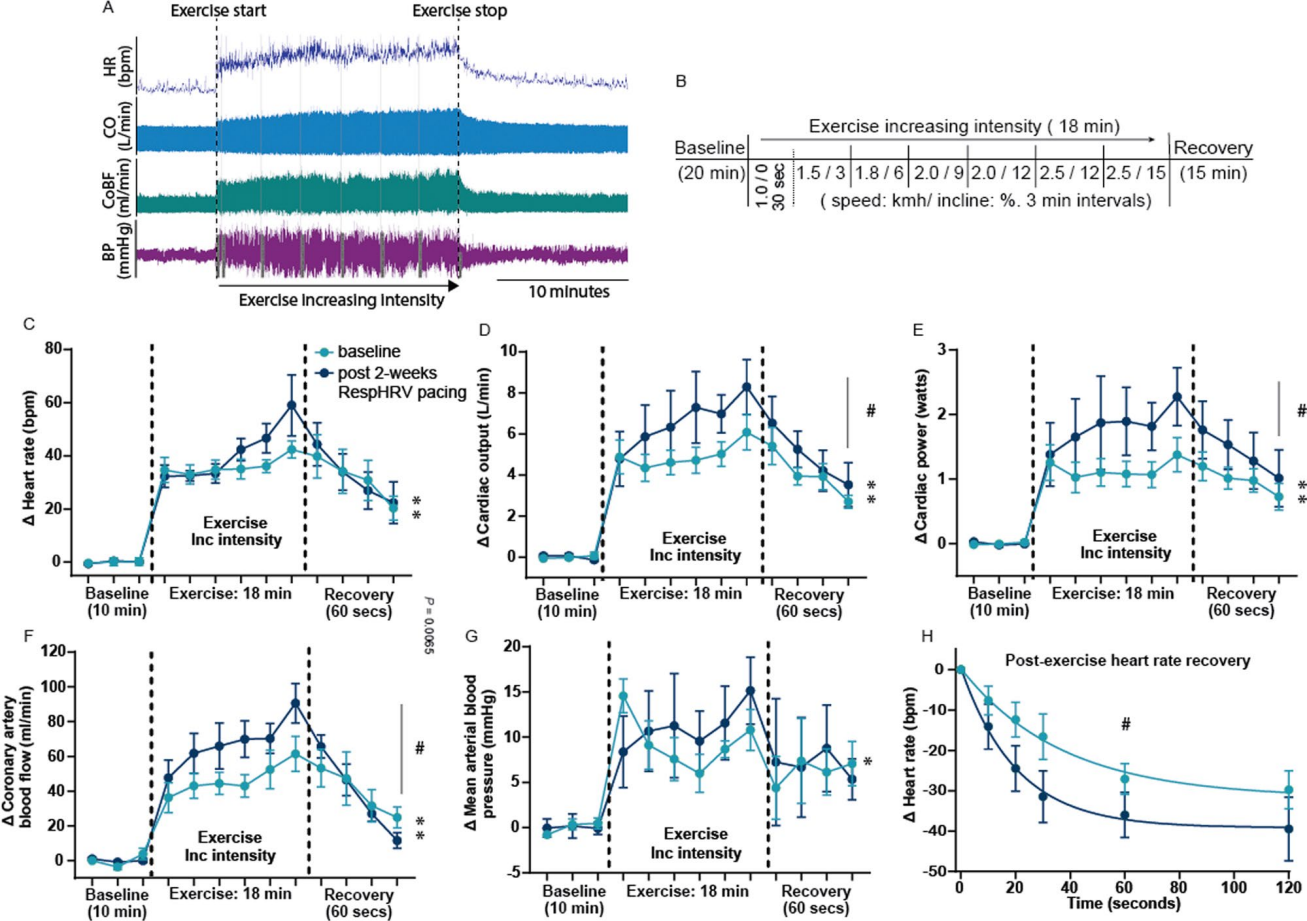
Hemodynamic responses to exercise in RespHRV-paced heart failure sheep. **A** Raw data trace of the hemodynamic exercise responses. **B**, A timeline of the graded exercise protocol. Change compared to baseline **C**, heart rate (baseline: *P = 4.1 × 10^–4^; post 2-week RespHRV -pacing: **P = 1.7 × 10^–3^. One-way ANOVA, effect of exercise, *n* = 6). **D**, cardiac output (baseline: *P = 2.6 × 10^–5^; post 2-week RespHRV pacing: **P = 1.6 × 10^–3^. One-way ANOVA, effect of exercise. # *P* = 3.7 × 10^–4^. Two-way ANOVA, Group effect, *n* = 6). **E**, cardiac power (baseline: *P = 2.4 × 10^–4^; Post 2-weeks RespHRV pacing: *P = 0.022. One-way ANOVA, effect of exercise. # *P* = 1.6 × 10^–4^. Two-way ANOVA, group effect, *n* = 5). **F**, coronary artery blood flow (baseline: *P = 4.1 × 10^–3^; post 2-weeks RespHRV pacing: * *P* = 6.6 × 10^–5^. One-way ANOVA, effect of exercise. # *P* = 1.7 × 10^–3^. Two-way ANOVA, group effect. † *P* = 0.078. Mixed-effects analysis, interaction effect, *n* = 5). **G**, Mean arterial pressure, (baseline only: *P = 0.019. One-way ANOVA, effect of exercise, *n* = 5) during exercise before (light blue) or after (dark blue) 2 weeks of RespHRV pacing. **H**, Heart rate recovery post-exercise (*n* = 6. # *P* = 0.046, paired t test). BP; blood pressure, CO; cardiac output, CoBF; coronary artery blood flow, HR; heart rate

Seven days after instrument implantation surgery, sheep were acclimatized to the treadmill exercise protocol for 3 days before the experimental exercise test, as previously described [52]. A previously optimized exercise protocol of an incrementing intensity to a maximum of 2.5 km/h with a 15% incline over 18 min was used for sheep either monotonically or RespHRV paced; in both cases, pacing was switched off during the exercise trials (Fig. 3B). The sheep walked freely with no negative reinforcement. At the end of the exercise session, the treadmill was switched off, and the animals were allowed to rest on the treadmill for 15 min for post-exercise recovery measurements. Exercise recovery measures were taken at 10-, 20-, 30-, 60, and 120-s post-exercise.

**Fig. 4:**
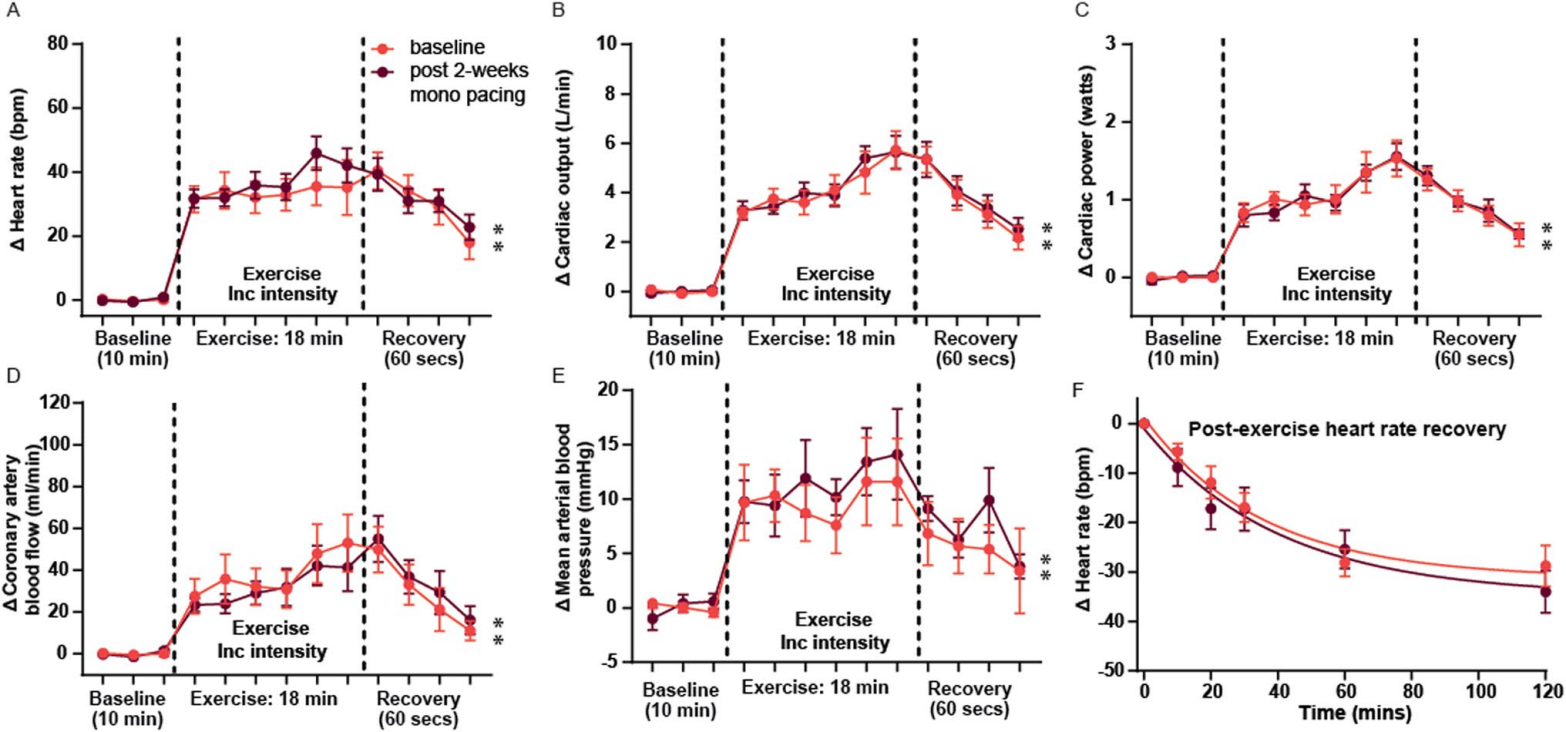
Hemodynamic responses to exercise in monotonically paced heart failure sheep. In stark contrast to RespHRV-paced sheep (see Fig. 3) **A**–**F**, no difference in any hemodynamic parameters during exercise before (red) or after 2 weeks of monotonic pacing (dark red) were observed. **A**, Heart rate (baseline: *P = 6.3 × 10^–7^; post 2-week mono-pacing: *P = 1.5 × 10^–9^. One-way ANOVA, effect of exercise, *n* = 8). **B**, Cardiac output (baseline: *P = 1.8 × 10^–5^; post 2-week Mono-pacing: *P = 7.4 × 10^–8^. One-way ANOVA, effect of exercise, *n* = 8). **C**, Cardiac power (baseline: *P = 7.9 × 10^–4^; post 2-week mono-pacing: *P = 1.0 × 10^–6^. One-way ANOVA, effect of exercise, *n* = 6). **D**, Coronary artery blood flow (baseline: *P = 0.014; post 2-week mono-pacing: **P = 3.5 × 10^–2^. One-way ANOVA, effect of exercise, *n* = 5). **E**, Mean arterial pressure (baseline: *P = 0.020; post 2-week mono-pacing: *P = 0.030. One-way ANOVA, effect of exercise, *n* = 6). **F**, Heart rate recovery, presented fitted with first-order exponential decay (*n* = 8)

**Fig. 5:**
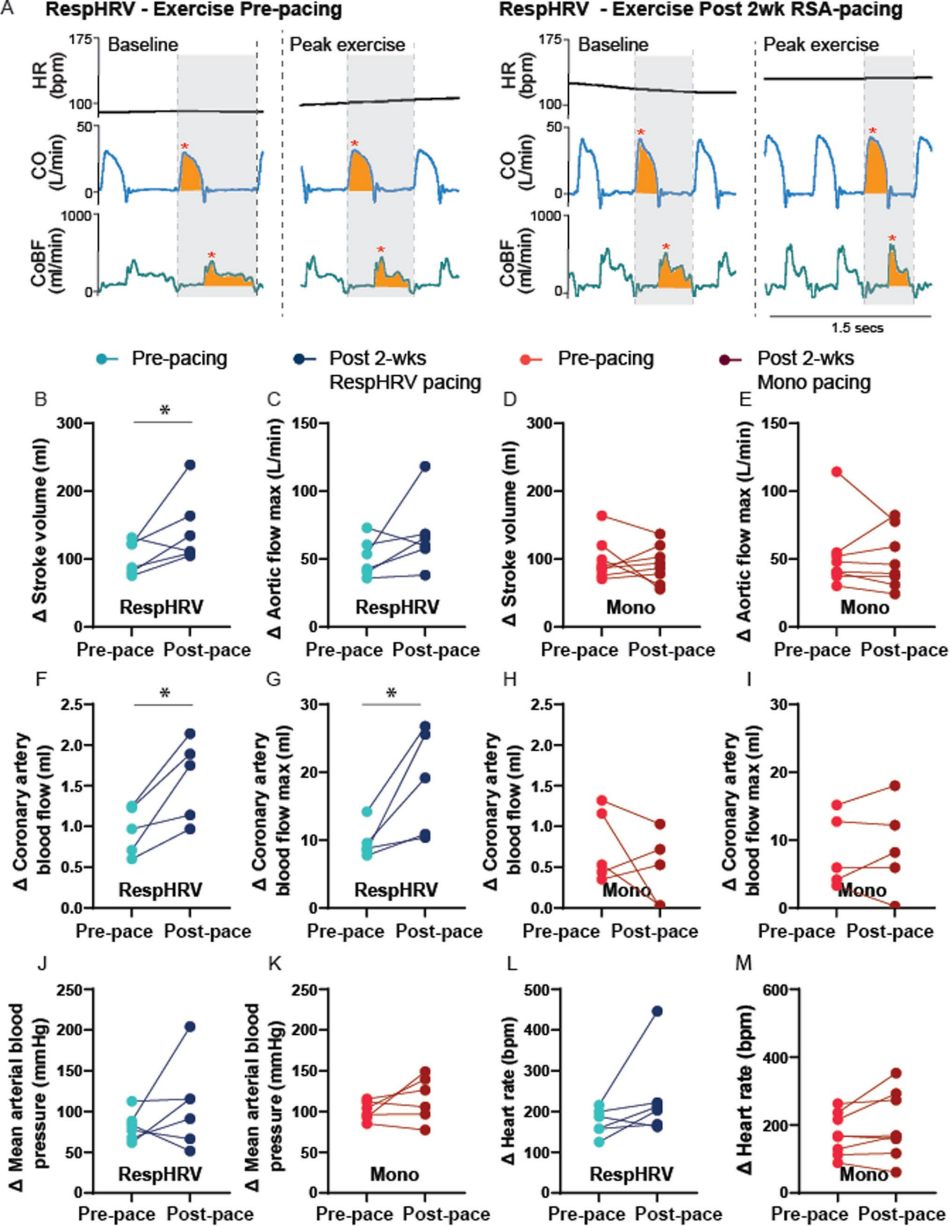
Single beat hemodynamics, normalized to heart period, during exercise. **A** Representative schematics of single-beat hemodynamics. Yellow highlighted area indicates area under the curve: cardiac output (CO) = stroke volume, coronary artery blood flow (CoBF) = total coronary blood flow per beat. Red asterisk indicates peak flow per beat. All data are normalized to heart period. B – M, Data points represent the change in a given parameter from baseline (stood on treadmill) to max exercise. Lighter circles represent the pre-pacing exercise test, with connecting lines to darker circles showing post-pace exercise test. **B** Change in stroke volume, normalized to heart period increases after RespHRV pacing (*P* = 0.025, paired t test), with no change in peak aortic flow **C**. D + E, No change in stroke volume of peak aortic flow after monotonic pacing. F + G, Total coronary artery blood flow, and peak coronary artery blood flow per beat increase more during exercise after RespHRV pacing (F: *P* = 0.017, G: *P* = 0.014, paired t test). H + I, With no change in coronary hemodynamics during exercise after monotonic pacing. J–M, Neither RespHRV nor monotonic pacing resulted in a change in the increase in mean arterial pressure or heart rate (HR) during exercise

Measurements of blood pressure, cardiac output, and coronary artery blood flow were recorded throughout the exercise protocol and presented as averages of 30 s at the end of each level of exercise (Figs. 3, 4), or exported as individual beats (using custom written scripts, Spike2), normalized to heart period, and averaged over 50 beats at baseline and maximum exercise (Fig. 5). For the individual beats, cardiac output and coronary artery blood flow were exposed as max flow per heartbeat, and area under the curve to measure stroke volume and coronary flow per beat. Due to experimental constraints and access to the treadmill, (RespHRV, *n* = 6) and (Mono, *n* = 8) underwent exercise testing. Although all sheep were implanted with all recording probes, not all sheep maintained signal in all probes over the entire experimental period (Table 1). All exercise data are paired pre-to post-pacing per sheep; data are only included when the signal is maintained throughout both exercise challenges (before and 2 weeks after pacing) (Table 1).

### Drug administration in Protocol B

In this protocol, following a baseline period of 5 days, the pharmacological agents were administered over the next 4 weeks continuously. A β-adrenergic receptor blocker (0.075 mg/kg/hr propranolol hydrochloride, AK Scientific, Inc. USA, Catalog No. J95387) and an AT_1_ receptor (AT_1_R) antagonist (0.08 mg/kg/hr losartan potassium, AK Scientific, Inc. USA, Catalog No.1934) were administered intravenously at a constant infusion rate of 1 ml/hr, 24 h a day for 4 weeks. Following 2 weeks of the drug protocol, RespHRV pacing was initiated and continued for 14 days while the drug infusion continued over the next 2 weeks (Table 2). The nonselective β-blocking agent, propranolol, was chosen given the negative chronotropic effects of propranolol which can be beneficial [27] while also reducing myocardial oxygen expenditure in heart failure [59].

**Table 2:** Raw values for resting hemodynamic parameters in conscious free-standing sheep that underwent the drugs + RespHRV protocol (Protocol B): 24-h averages

	Baseline (not paced)	2 weeks of drug infusion (not paced)	4 weeks of drug infusion and 2 weeks of RespHRV pacing (paced)
Heart rate (bpm) RespHRV, *n* = 6	100 ± 7.5	93 ± 7.3	118 ± 2.7
Cardiac output (L/min) RespHRV, *n* = 6	8.4 ± 0.9	8.9 ± 0.8	10.1 ± 0.7
Coronary artery blood flow (ml/min) RespHRV, *n* = 6	80 ± 15	67 ± 16	77 ± 16
Mean arterial pressure (mmHg) RespHRV, *n* = 6	86 ± 2.9	78 ± 6.5	70 ± 6.4

### Statistical analysis

All data are expressed as mean ± SEM, except where indicated. All time course data (cardiac output, coronary artery blood flow, heart rate, mean arterial pressure) were analyzed between groups using a repeated measures 2-way ANOVA, or mixed-effects model if any missed data points were 'missing at random'. The effect of time (or exercise) within group monotonic or RespHRV paced was analyzed using a one-way ANOVA. Total peripheral resistance (SVR mmHg/L/min) was calculated by mean arterial pressure/cardiac output, and coronary vascular resistance (CoVR mmHg/ml/min) was calculated by mean arterial pressure/coronary artery blood flow. Cardiac power output, used as a measure of energy output or work done by the heart, was calculated using the equation mean arterial pressure x cardiac output/451 as previously described [18, 33]. Heart rate recovery post-exercise was fitted with a first-order exponential decay with statistics presented as the time constant Tau using a paired t test [44] in Microsoft Excel. All other statistical analyses were performed in SPSS (v8.1)**.** Data were considered significant if *P* < 0.05.

## Results

### Reinstating RespHRV in heart failure (Protocol A)

There were no differences in baseline ejection fraction before the induction of heart failure (microembolization) (RespHRV: 63.9 ± 3.4%. Mono: 66.7 ± 3.6%) or 8–10 weeks later, after the establishment of chronic heart failure and before the start of the experiments (RespHRV: 45.8 ± 3.4%. Mono: 43.0 ± 3.7%) between groups (experimental timeline Fig. 1).

Hemodynamic measures, including arterial pressure, cardiac output, coronary artery blood flow, and heart rate, were recorded for 24 h a day for 4 weeks in conscious sheep (Fig. 1). Reinstatement of RespHRV for 2 weeks in fully instrumented conscious sheep with chronic heart failure increased cardiac output (Δ 1.65 ± 0.2 L/min, *n* = 6, mean ± SEM) from baseline; in contrast, there was no change in cardiac output in sheep paced monotonically (-0.18 ± 0.5 L/min, *n* = 8, mean ± SEM) (Fig. 2A). Both groups were paced to the same mean heart rate (Fig. 2B), showing that the increase in cardiac output in the RespHRV-paced group was not dependent on a difference in the number of heartbeats between groups. In RespHRV-paced sheep, the cardiac output increased after, on average (median), days 2.5 of pacing, and this climbed steadily over the first 5–7 days (Fig. 2A and supplemental Fig S1A). Interestingly, the increase in cardiac output was not associated with a change in coronary artery blood flow or coronary vascular resistance throughout the RespHRV pacing. RespHRV pacing did not affect breathing rate as previously reported [51]. Circadian modulation of cardiac output was maintained in both RespHRV and monotonically paced groups (Supplemental Fig. S1). There were no significant changes in any of the other variables in the monotonic pacing group. There was no statistical difference between mean arterial pressure, systemic vascular resistance, coronary artery blood flow, or coronary vascular resistance between groups over time (Fig. 2C–F). Pacing efficacy (percentage of the day sheep were paced) was 35 ± 6.7% at 1 week, 40 ± 8.2% at 2 weeks in RespHRV paced and 87 ± 5.1% at 1 week, and 91 ± 2.8% at 2 weeks in monotonically paced sheep.

### Cardiovascular responses to exercise in heart failure before and after RespHRV pacing

Hemodynamic parameters from conscious sheep were recorded during a graded exercise protocol (Fig. 3A + B). There were no differences in any of the hemodynamic changes during exercise between the RespHRV-paced and monotonic-paced groups at the pre-pacing (baseline) time point. After 2 weeks of RespHRV pacing, sheep exhibited comparable heart rate changes during exercise compared to before pacing (Fig. 3C). However, after 2 weeks of RespHRV pacing, sheep exhibited greater increases in cardiac output, coronary artery blood flow and cardiac power output during exercise, compared to pre-pacing (difference baseline + post-pacing: *P* < 0.001. Figure 3D–F), indicating increased cardiac function and efficiency during moderate exercise. There was a comparable increase in mean arterial pressure (Fig. 3G), and systemic vascular resistance (Supplemental Fig S2) before and after pacing. Interestingly, RespHRV pacing increased post-exercise heart rate recovery compared to the pre-pacing baseline value (*P* < 0.05, Fig. 3F). One sheep in the RespHRV-paced group did not complete the full exercise protocol at baseline (completed up to and including level 5 of 6); however, this sheep went on to complete the whole protocol after 2 weeks of RespHRV pacing.

### Cardiovascular responses to exercise in heart failure before and after monotonic pacing

At baseline (after instrumentation surgery and before cardiac pacing) and 2 weeks after monotonic pacing, there was no difference in any parameters measured during exercise in the monotonically paced group (Fig. 4A–E), and no change in the rate of heart rate recovery post-exercise (Fig. 4F). In the monotonically paced group, one sheep did not complete the full exercise protocol at baseline (completed up to and including level 4 of 6). This animal and one additional animal did not complete the full exercise protocol after 2 weeks of monotonic pacing (both sheep completed up to and including level 5 of 6).

### Single beat dynamics during exercise

Given the beat-to-beat measures of variables, we compared the change in single-beat aortic flow dynamics (normalized to heart period) during baseline (Pre-pace) exercise (Fig. 4A) to change in single-beat aortic flow dynamics during exercise after RespHRV pacing (post-pace) (Fig. 4B). RespHRV pacing resulted in a greater increase in stroke volume per beat during exercise (Fig. 5B) but no difference in the peak aortic flow per beat (Fig. 5C). Both total coronary flow and max coronary flow increased by a greater amount during exercise after RespHRV pacing (Fig. 5F + G). There was no difference in the change in single-beat cardiac output or coronary flow dynamics during exercise after cardiac pacing in the mono-paced group (Fig. 5D + E, H + I). Mean arterial pressure and heart rate changes during exercise were not different in either paced group (Fig. 5J-M).

### Reinstatement of RespHRV in heart failure with current medications (Protocol B)

**Fig. 6:**
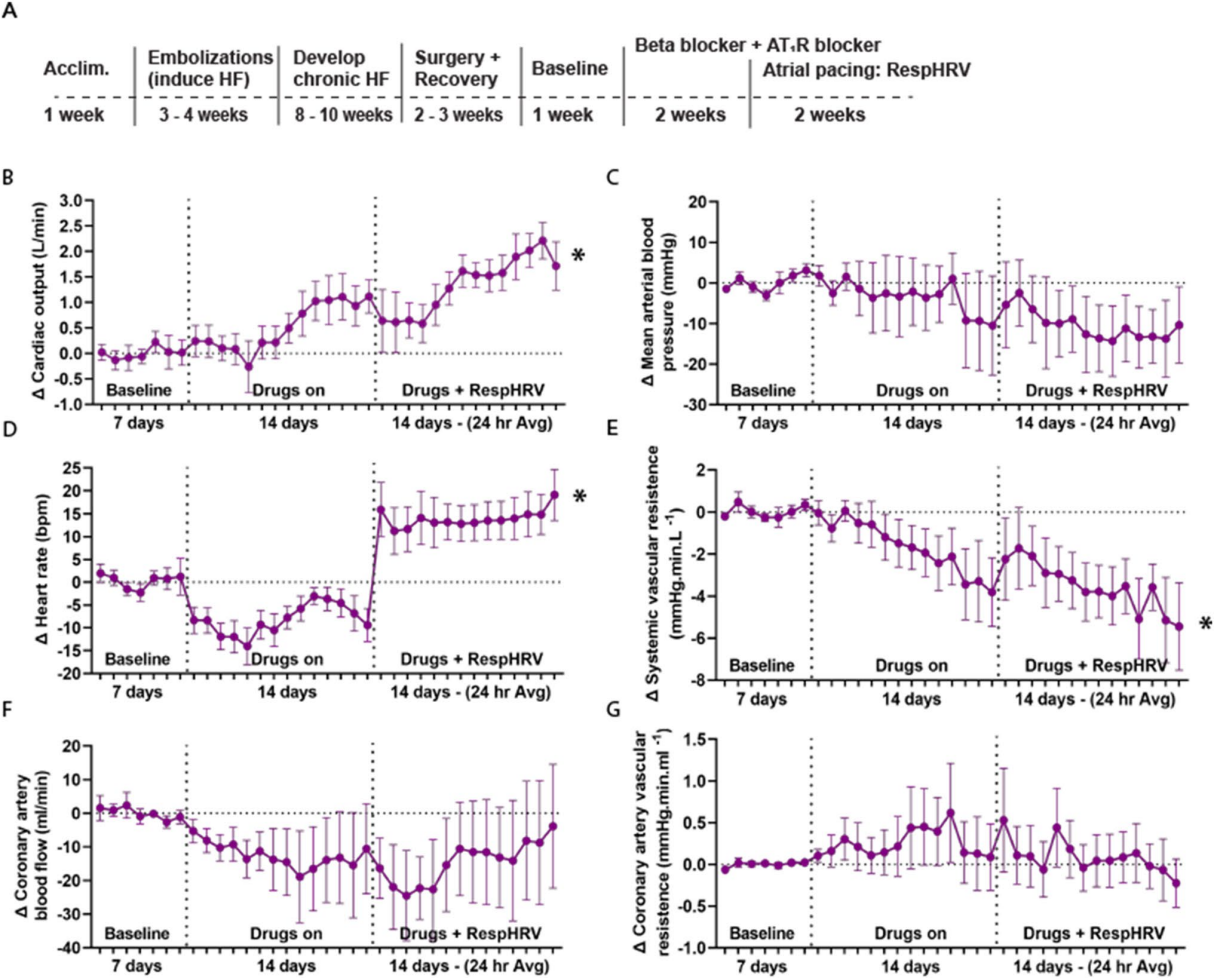
Experimental set-up and 5-week drugs with RespHRV protocol. **A**, A modified timeline from Fig. 1A showing experimental protocol in drug animals. **B**, Two weeks of RespHRV pacing on the background of drugs (*n* = 6) significantly increased cardiac output (*P = 7.5 × 10^–14^, one-way ANOVA, effect of time), D, heart rate over time (*P = 3.9 × 10^–28^, one-way ANOVA, effect of time, drugs for 4 weeks, drugs + paced for 2 weeks), E, systemic vascular resistance (*P = 3.7 × 10^–9^, one-way ANOVA, effect of time). No change in C, mean arterial pressure (*n* = 6), F, coronary artery blood flow (*n* = 6), or G. coronary artery vascular resistance (*n* = 6). B-G, Each data point is a 24-h average

After baseline recordings, constant intravenous infusions of a combined β-blocker (Propranolol) and an AT_1_R blocker (Losartan) for 14 days before RespHRV pacing resulted in a significant decrease in heart rate and a significant increase in cardiac output (Fig. 6B + D). The increase in cardiac output was not associated with a change in coronary artery blood flow or coronary vascular resistance over the 14 days of the medications (Fig. 6F + G). To confirm that the AT_1_R blocker was given at an effective dose to block the AT_1_ receptor, the pressor response to intravenous angiotensin II was tested as previously described [54]. AT_1_R blockade was tested at baseline before the administration of the AT_1_R blocker, after the administration of the AT_1_R blocker, and at the end of the study. The angiotensin II-mediated pressor response was significantly reduced after administration of an AT_1_R blocker compared to baseline (Supplemental Fig S3). The decreased resting heart rate indicated an effective blockade of the β-adrenergic receptors. Previously published heart failure ‘time-control’ experiments using the same chronic recordings show no reduction in heart rate over time [51]. Reinstatement of RespHRV, against this background of medications, further increased cardiac output (Δ 1.33 ± 0.2 L/min from the start of RespHRV pacing, *n* = 6, mean ± SEM) in heart failure. However, the time course of onset was slower compared to when no drugs were on board (day 2.5 of pacing in RespHRV-paced sheep without medication versus day 6 on RespHRV-paced sheep with medications on board) (Supplemental Fig S2). Systemic vascular resistance showed a significant decrease (*P* < 0.001, Fig. 6.E). No statistically significant changes were observed in mean arterial pressure, coronary artery blood flow, or coronary vascular resistance in RespHRV-paced sheep with medications over time (Fig. 6B, F, G).

## Discussion

Our study presents four major novel findings on the clinical benefits of reinstating physiological RespHRV in heart failure. *First*, RespHRV pacing causes no change in coronary artery blood flow at rest, while cardiac output increased in a completely novel set of animals to those reported previously [51]. *Second*, during exercise, RespHRV pacing improved cardiac output, coronary artery blood flow, and cardiac power output, independent of changes in heart rate compared to pre-pacing. *Third*, post-exercise heart rate recovery is improved after RespHRV pacing. *Fourth*, against a background of a β-blocker and an AT_1_R blocker, RespHRV pacing *further* increased cardiac output with no change in coronary artery blood flow at rest. Taken together, our findings suggest new generation pacemakers should incorporate RespHRV.

### Effect of RespHRV pacing on coronary artery blood flow at rest

The relationship between cardiac output and coronary artery blood flow is complex, influenced by metabolic factors, autoregulation, and endothelial function [28]. In chronic ischemic heart disease, this relationship is impaired [26], contributing to both the cause and progression of cardiac dysfunction [26]. We anticipated that the increase in cardiac output with RespHRV pacing would be accompanied by increased coronary artery blood flow. However, after 2 weeks of RespHRV pacing, there was no change in coronary flow or vascular resistance at rest suggesting improved cardiac efficiency rather than enhanced oxygen delivery. Our previous mathematical modeling data, which proposed that the functional role of RespHRV is to improve cardiac efficiency, aligns with these findings [8]. The mechanism behind RespHRV-induced efficiency gains remains unclear but may involve mitochondrial function, cell-to-cell communication [16, 36], or myocyte structure restoration [14, 51].

### Effect of RespHRV pacing on exercise capacity in heart failure

The most life-limiting symptom of living with heart failure is reduced exercise tolerance. During exercise in healthy individuals, cardiac output increases to meet the increased metabolic demands of the body, including the myocardium [35] and this cardiac output increase is one of the primary determinants of exercise capacity [9]. While exercise intolerance is multifactorial in patients with heart failure, the inability to sufficiently increase cardiac output during exercise directly correlates to reduced exercise capacity and worsening quality of life [9, 46]. Following 2 weeks of RespHRV pacing, cardiac output was not only increased at rest but was further elevated during moderate exercise. The increase in cardiac output during exercise was due to an increase in stroke volume, independent of heart rate changes. Moreover, beat-to-beat stroke volume increased during exercise after RespHRV pacing, but the maximum aortic flow per beat did not. These results suggest that an increase in the peak force of contraction is not driving the increase in cardiac output during exercise, but rather increased diastolic function and ventricular filling. The total increase in cardiac output during exercise was ~ 35% greater after RespHRV pacing; this compares to ~ 20% increase in cardiac output with current best use pacemakers [50].

Although no change in resting coronary flow was observed after RespHRV pacing at rest, 2 weeks of RespHRV pacing resulted in a significant increase in coronary blood flow during exercise. This increase in coronary blood flow during exercise after RespHRV pacing was observed as an increase in maximum and total flow per beat (normalized to heart period). The enhanced ability to increase total beat-to-beat coronary blood flow from rest suggests increased coronary flow reserve after RespHRV pacing [53]. As maximum coronary flow occurs during diastole, the increased maximum coronary flow per beat further indicates improved diastolic function during exercise after RespHRV pacing. This is similar to changes observed with CRT pacing, where total coronary flow at rest is not altered but there is an increase in coronary flow reserve to a hyperaemic challenge [19, 31, 34].

In addition to the changes in flow dynamics, 2 weeks of RespHRV pacing resulted in an increase in cardiac power output during exercise. Maximum cardiac power output has been shown to strongly correlate with exercise capacity [13], and VO_2max_ [6]. In patients with chronic heart failure, the ability to increase cardiac power output during exercise is a strong predictor of adverse cardiac outcomes [11, 58, 60] and mortality [58]. Our data indicate that RespHRV pacing can augment increases in cardiac power output and may, therefore, prolong life in heart failure.

All the beneficial effects of 2 weeks of RespHRV pacing on cardiac function during exercise were observed while the pacemaker was turned off (the pacemaker was always off when the sheep was on the treadmill). We have previously reported myocyte remodeling after RespHRV pacing and that the resting cardiac output takes up to 7 days to return to baseline after the pacemaker is turned off [51], indicating that RespHRV pacing induces benefits that exist beyond the pacemaker being switched off. Current pacing strategies to increase heart rate during activity, rate-adaptive pacing, have not shown any benefits in increasing exercise capacity [10], suggesting a novel improvement in exercise capacity with restoration of ResHRV pacing.

### Effect of RespHRV pacing on heart rate recovery

RespHRV pacing improved post-exercise heart rate recovery, indicating better autonomic balance. Reduced parasympathetic nerve activity in heart failure is linked to slower heart rate recovery after exercise [21, 43], predicting poor cardiovascular outcomes [29], hospitalizations [5], and death [38]. Two weeks of RespHRV pacing led to faster heart rate recovery post-exercise, suggesting increased parasympathetic nervous system responsiveness. Studies in control rats [32] and sheep [52] have recently shown that increased parasympathetic activity during exercise benefits cardiac function and increases coronary blood flow. It is tempting to speculate that the improved cardiac function may be mediated, in part, by restoration of parasympathetic activity, though this requires further evaluation.

### Effect of β-blocker and AT_1_R blocker together with RespHRV pacing on heart function

In Protocol A, sheep with heart failure were not on any common heart failure medications, unlike the expected clinical population. To further establish the benefits of RespHRV pacing alongside these medications, we conducted a separate protocol with HF sheep on a combined β-blocker and AT_1_R blocker therapy (Protocol B). While 2 weeks of administration of these drugs alone improved cardiac output, it was to a lesser extent than RespHRV pacing alone (in Protocol A). Importantly, reinstatement of RespHRV pacing, while sheep were still receiving the β-blocker and AT_1_R blocker, resulted in a further increase in cardiac output. These results indicate that the improvement in cardiac output was in addition to the benefit of the medications. There was no change in resting coronary artery blood flow with either medications alone or medications with RespHRV pacing. An important point that warrants further consideration is that the increase in cardiac output appeared to be delayed compared to when no medications were on board (day 6 versus day 2.5). The reasons for the delay are unclear, but important to be aware of before initiating large-scale clinical trials.

## Study limitations

Only female sheep are used in this study. Therefore, sex as a biological variable cannot be assessed. Coronary artery blood flow was measured using a Doppler flow probe chronically implanted around the left coronary artery. Therefore, we cannot draw any conclusions about changes in epicardial to endocardial blood flow, which may be important in cardiac function in heart failure. The microembolization model of HFrEF was chosen based on our previous experience with developing a consistent model of heart failure [3] while adhering to animal welfare principles. We acknowledge that microembolization does not fully replicate acute heart failure resulting from acute myocardial infarction. This model has been used by multiple groups to produce heart failure whereby the microvascular obstruction and dysfunction result in patchy microinfarcts contributing to progressive myocardial contractile dysfunction [22, 30, 48]. This model simulates key features of HFrEF, including impaired heart rate variability and autonomic imbalance making it an ideal platform to study the effects of modulating RespHRV. Our study used the nonselective β-blocking agent, propranolol. We acknowledge that the effects of more recent cardio-selective beta-blockers together with RespHRV pacing needs to be investigated.

## Clinical implications

Cardiac device development is a rapidly advancing field, shifting beyond the traditional view of the heart as a simple pump towards integrating physiological mechanisms that regulate cardiac function. Pacemakers developed in the last 2 decades include chamber synchronization [19, 50, 55], cardiac contractility modulation [2], and rate-adaptive pacing [10] to reflect this. To date, none of the commercial pacemakers incorporate RespHRV and our study highlights the enormous potential of reinstating RespHRV alongside standard heart failure medications. While our preclinical study used diaphragmatic electrodes as a stable respiratory sensor in conscious sheep, we anticipate clinical RespHRV devices to be integrated with nasal thermocouples or chest wall impedance sensors.

## Conclusion

Cardiovascular fitness is a key predictor of overall morbidity and mortality [47], with exercise intolerance being a primary symptom of patients with heart failure. Novel treatments are needed urgently. Here, we show that reinstating RespHRV in heart failure increases cardiac output without changing coronary artery blood flow at rest and improves exercise tolerance. These findings indicate that restoring RespHRV may improve cardiac efficiency and as well as exercise capacity in heart failure. Importantly, these beneficial effects of RespHRV pacing persist with medications on board. Our study emphasizes the importance of implementing RespHRV restoration in clinical trials for individuals living with heart failure.

## Supplementary Information

Below is the link to the electronic supplementary material.

Supplementary file 1 (DOCX 878 KB)

## Abbreviations

DEMG: Diaphragmatic electromyograph
Mono: Monotonic
RespHRV: Respiratory heart rate variability
