# Supplementary material for: Reinstating respiratory heart rate variability improves hemodynamic responses during exercise in heart failure with reduced ejection fraction
Source: Basic Res Cardiol. 2025 May 3;120(4):745–59. doi: 10.1007/s00395-025-01110-3 (PMC12325447; doi:10.1007/s00395-025-01110-3)
Supplement: Supplementary file 1 — (DOCX 878 KB) [file 395_2025_1110_MOESM1_ESM.docx]

**Supplemental Figure S1:**

**
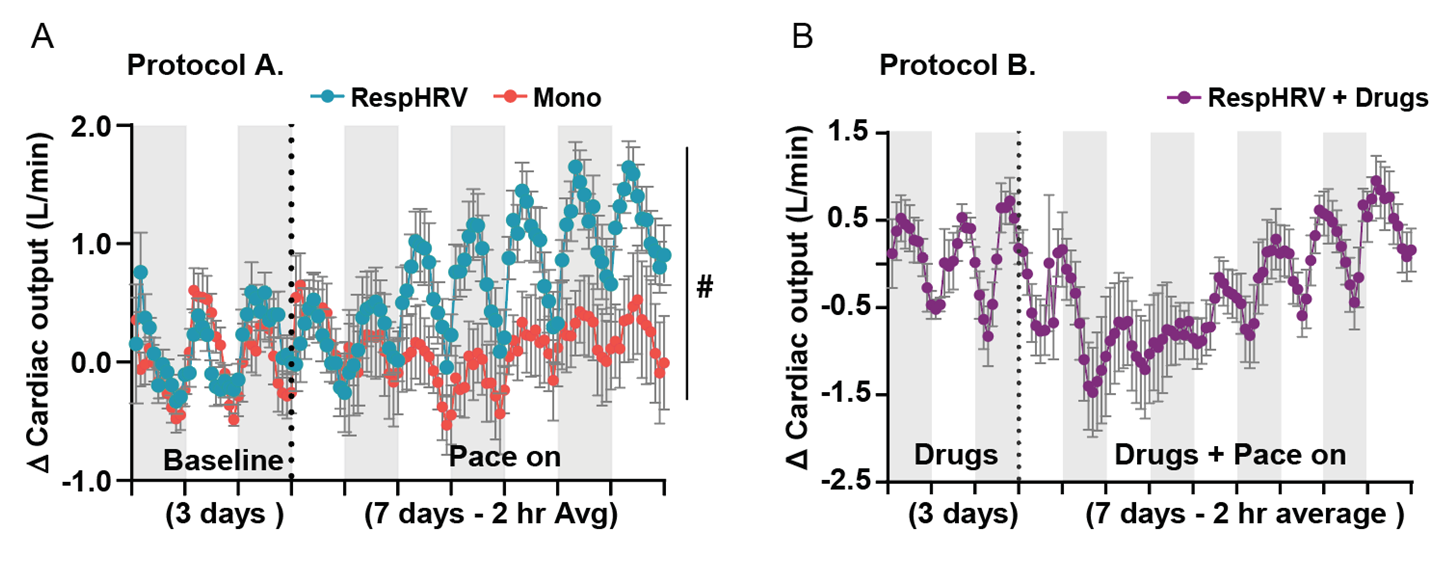
**

**Supplemental Figure S1: Circadian variation in resting cardiac output**

A, RespHRV pacing significantly increased cardiac output over 7 -days, note circadian pattern in B, (# P = 3.16 x10^-14^ , Two-way ANOVA, interaction effect) . B, During concurrent RespHRV pacing and heart failure medication, cardiac output increased more slowly. Each data point is a 2-hr average.

**Supplemental Figure S2:**

**
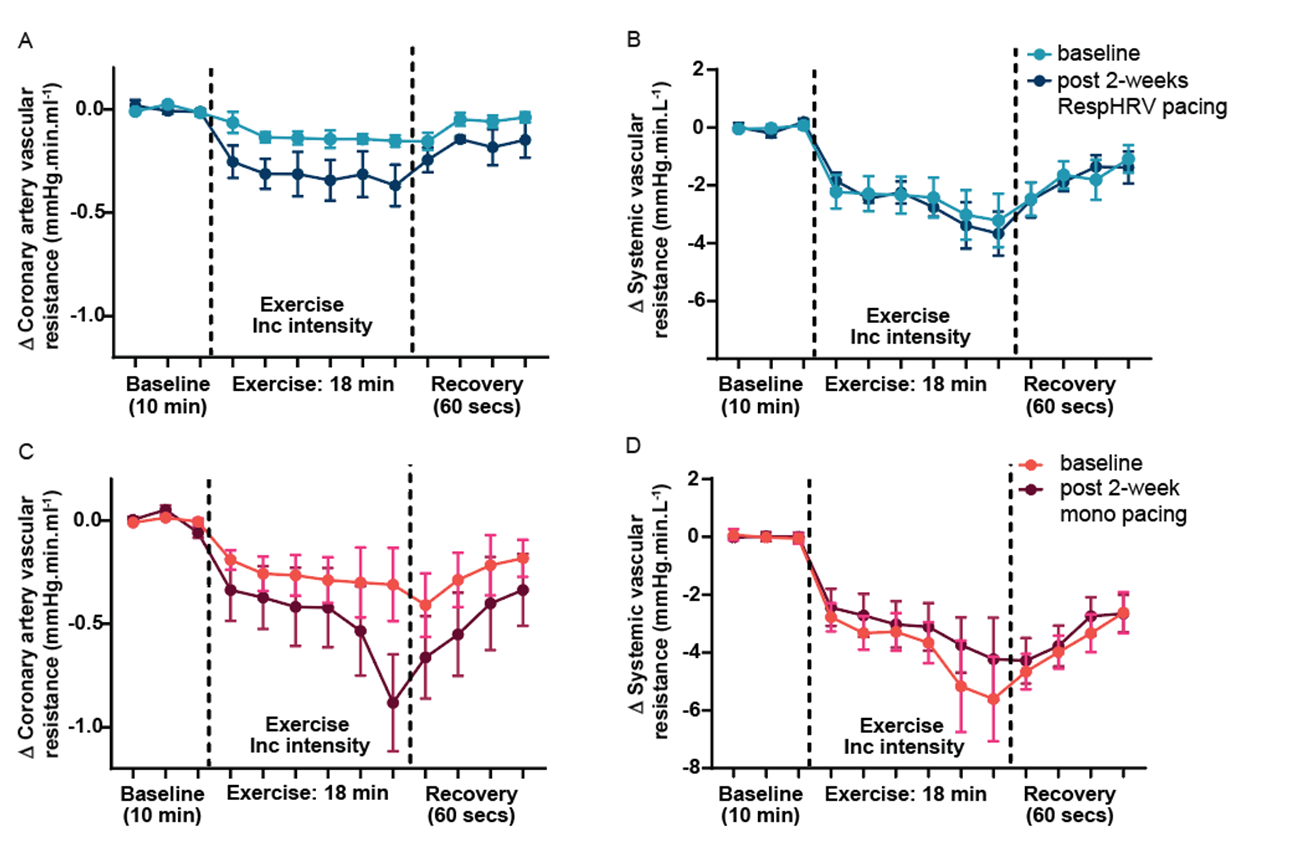
**

**Supplemental Figure S2: Vascular resistance changes during exercise**

A, RespHRV pacing; Change in coronary artery vascular resistance during exercise (n = 4, no statistical tests on low n), and B, post-RespHRV pacing no change in systemic vascular resistance during exercise compared to baseline (n = 5). C, monotonic pacing; coronary vascular resistance (n = 4). D, no change in systemic vascular resistance ( n = 6) during exercise after 2-weeks monotonic pacing.

**Supplemental Figure S3:**

**
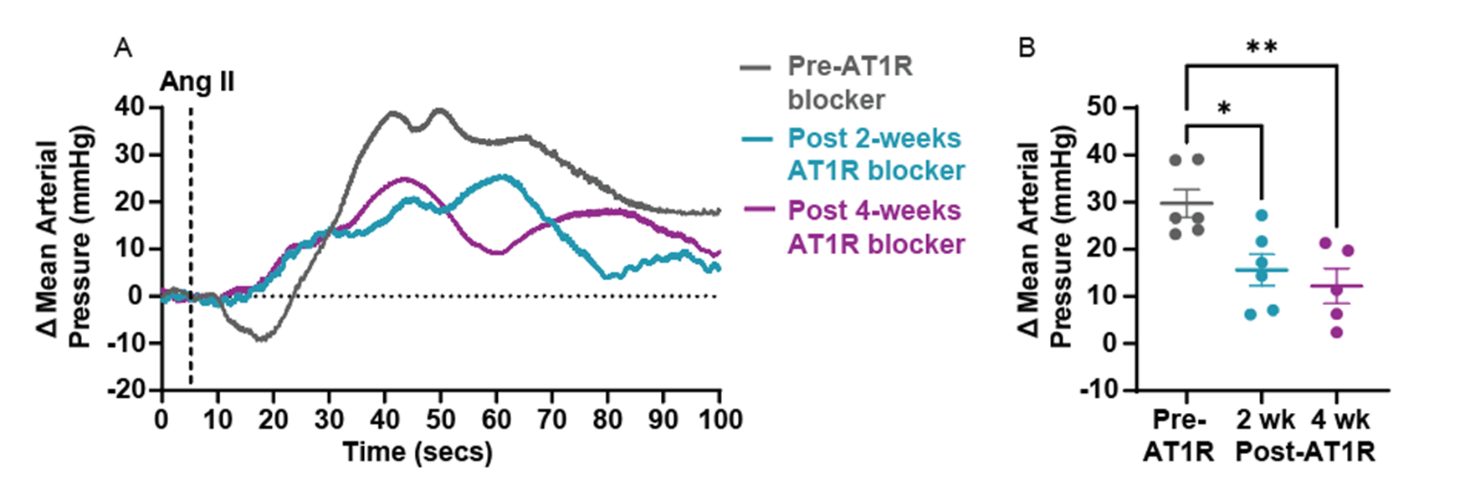
**

**Supplemental Figure S3: Pressor response to AngII challenge.**

The increase in mean arterial pressure after administration of intravenous AngII was reduced after AT1R blockade. A, representative raw data. B, Group data, data points represent individual animals (** P < 0.005, * P < 0.05 one-way ANOVA. )
